# Colour-Difference Measurement Method for Evaluation of Quality of Electrolessly Deposited Copper on Polymer after Laser-Induced Selective Activation

**DOI:** 10.1038/srep22963

**Published:** 2016-03-10

**Authors:** Mindaugas Gedvilas, Karolis Ratautas, Elif Kacar, Ina Stankevičienė, Aldona Jagminienė, Eugenijus Norkus, Nello Li Pira, Gediminas Račiukaitis

**Affiliations:** 1 Center for Physical Sciences and Technology, Savanoriu Ave. 231, LT-02300 Vilnius, Lithuania; 2 Kocaeli University, Faculty of Arts and Sciences, Department of Physics, Umuttepe Campus, 41380, Kocaeli, Turkey; 3 Group Materials Labs, Centro Ricerche Fiat S.C.p.A., Strada Torino 50, 10043 Orbassano (TO), Italy

## Abstract

In this work a novel colour-difference measurement method for the quality evaluation of copper deposited on a polymer is proposed. Laser-induced selective activation (LISA) was performed onto the surface of the polycarbonate/acrylonitrile butadiene styrene (PC/ABS) polymer by using nanosecond laser irradiation. The laser activated PC/ABS polymer was copper plated by using the electroless copper plating (ECP) procedure. The sheet resistance measured by using a four-point probe technique was found to decrease by the power law with the colour-difference of the sample images after LISA and ECP procedures. The percolation theory of the electrical conductivity of the insulator conductor mixture has been adopted in order to explain the experimental results. The new proposed method was used to determine an optimal set of the laser processing parameters for best plating conditions.

Polymer-based electrical devices are used in different areas such as optical communications, biomedicine, and automotive industry^1^,^2^. Formation of the electrical circuits on polymers is important for these devices. There are many techniques called “selective metallization” to produce a circuit on the surface of polymers: direct chemical plating^3^, laser direct structuring (ablation and writing)^4^,^5^, laser direct imaging^6^ and laser-induced selective activation (LISA)^7^,^8^,^9^,^10^,^11^.

LISA was developed for selective metallization of polymers for three-dimensional moulded interconnecting devices. This method contains single step: laser structuring of the polymer surface. As a result of the laser structuring, sponge-like and nano-porous structures are created to improve the adhesion strength of the metal to the polymer. After LISA process the metal layer is deposited by the electroless copper plating (ECP) procedure. The ECP contains of three-steps: activation of the laser-structured surface with palladium colloidal activator; cleaning with distilled water; electroless deposition of copper layer on the activated sample. In the activation procedure, palladium atoms are trapped in a porous sponge like structures on the polymer surface. Cleaning with distilled water removes unwanted palladium from flat unstructured polymer and leaves it confined in the poriferous surface. The copper fills all the openings in the polymer and a porous, rough metal film is deposited during the final step of the ECP. Finally, after both, LISA and ECP procedures, the copper layer is deposited selectively only onto the laser structured areas while unstructured polymer is not coated with the metal deposition.

The electrical and optical properties of the semi-transparent films were investigated in numerous works^12^,^13^,^14^,^15^,^16^,^17^,^18^,^19^. However, the analysis of the sheet resistance dependence on the optical transmittance can be applied only for films with the thicknesses smaller than the absorption depth. Such kind of measurements cannot be realized with a relatively thick metal layer on the porous opaque substrate.

The characterization of the cross section of the metal plated polymer by the LISA and ECP procedures has been performed in the previous work by Y. Zhang *et al.*^7^. The bearing ratio versus depth of the porous surface has been introduced in the later works of the same group^9^,^11^. It was shown that there is no strict metal/polymer boundary and the thickness of non-uniform copper deposition on the sponge like polymer can not be measured by the standart methodologies: thickness profilometry, spectral reflectance, ellipsometry, interferometry, atomic force microscopy, etc.

In the last decade, the scientific works exploring the colour change upon the thickness of a material emerged. A number of graphene layers were measured according to its colour change by Y.-F. Chen *et al.*^20^. The colour variation as a function of the ceramic thickness was found by G. B. de Azevedo Cubas *et al.*^21^. The effect of colour on the thickness of the enamel porcelain was found by F. D. Jarad *et al.*^22^. The thickness of the lubricant film was measured by colorimetric interferometry by M. Hartl *et al.*^23^. The thickness profile was measured using the interference colour analysis by K. Kitagawa *et al.*^24^. A very high optical contrast upon switching between the amorphous and crystalline phases depending on the film thickness was demonstrated by P. Hosseini *et al.*^25^. The colour change of an ultra-thin highly absorbing film depending on the thickness because of the interference effect was demonstrated by M. A. Kats *et al.*^26^. However, in all above mentioned publications, the colour variation depending on the thickness was investigated for ultra-thin flat films and, therefore, cannot be applied to the porous, rough surfaces. The main idea proposed in this work is to evaluate the amount of a rough metal film plated on the sponge-like polymer surface by measuring the colour difference of the sample before and after metal plating.

In this work, the new colorimetric method for determining the sheet resistance of the plated copper and evaluating the plating quality is proposed. A polycarbonate/acrylonitrile butadiene styrene (PC/ABS) polymer was structured by the LISA method and then plated with copper by the ECP procedure. The sheet resistance measured by using the four-point probe technique was found to decrease by the power law with increasing the colour difference of sample images before and after copper deposition. The percolation model of the electrical conductivity of isolator-conductor mixture has been adopted to explain the experimental results. The new proposed method was used to determine an optimal set of laser processing parameters for the best metal plating conditions.

## Results and Discussion

### LISA and ECP procedures

**Figure 1:**
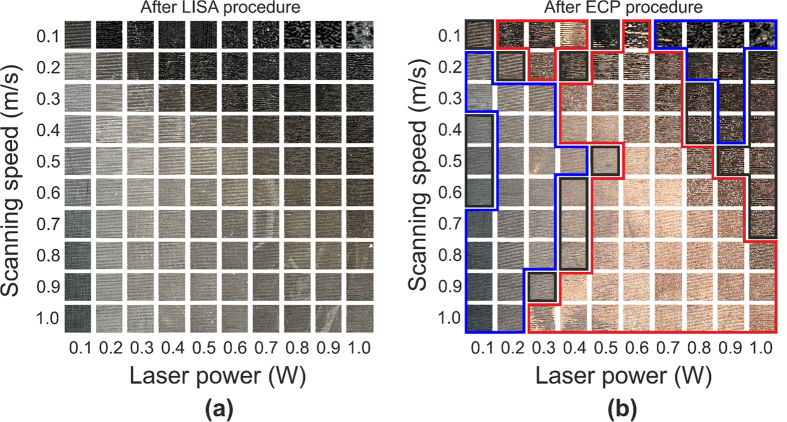
Images of the polymer before and after copper plating. Optical microscope images of PC/ABS polymer surface: (**a**) after LISA procedure - before copper plating; (**b**) after ECP procedure - after copper plating. The regions in (**b**): red solid line – sheet resistance of 0.1 Ω/sq < *R*_s_ < 1 Ω/sq; black solid line −1 Ω/sq < *R*_s_ < 10 Ω/sq; blue solid line −10 Ω/sq < *R*_s_ < 2 × 10^4^ Ω/sq. The size of each square image is 1 × 1 mm^2^.

Both LISA and afterwards ECP procedures were performed on the surface of the PC/ABS polymer sample. The optical microscope images of the samples after the LISA procedures are given in Fig. 1a. The LISA procedure induced the colour change and porous surface roughening on the polymer depending on the laser processing parameters applied. Later, after the LISA procedure, samples were processed by the ECP procedure. The layer of copper was deposited on the polymer areas with different thicknesses and morphologies depending on the laser processing parameters applied before. The images of the samples after the ECP procedure are given in Fig. 1b. The measured sheet resistances of the copper plated polymer varied from 0.1 Ω/sq to 2 × 10^4^ Ω/sq. The regions marked with the red solid line indicates well-plated areas with the sheet resistance of 0.1 Ω/sq < *R*_s_ < 1 Ω/sq (Fig. 1b). The areas with the moderate plating characteristics are marked by the black solid line with the sheet resistance of 1 Ω/sq < *R*_s_ < 10 Ω/sq (Fig. 1b). The blue solid line indicated areas represent the poor copper deposition with the sheet resistance of 10 Ω/sq < *R*_s_ < 2 × 10^4^ Ω/sq (Fig. 1b).

The quality of copper plating from the visual analysis of the pictures in Fig. 1b depended on both processing parameters: the laser power and the scanning speed. In the previous studies of ECP, for some particular plating conditions the extremely rough surface with the surface roughness factor (i.e. ratio between real surface area and geometrical surface area) of up to 124 was produced by E. Norkus *et al.*^27^ depending on the plating procedure applied^28^. That can change the natural colour of copper producing a black surface. However, in our case, the ECP procedure did not change the colour of copper. The average measured red green blue (RGB) colour components was of (0.686, 0.470, 0.196). The natural natural copper colour with RGB components of (0.722, 0.451, 0.200) was reported by A. Maerz *et al.*^29^. Only small colour difference of Δ*E* = 0.04 was calculated by comparing colours of our deposition and natural copper.

The measured minimal sheet resistance of the best plated area of *R*_s min_ = 0.1 Ω/sq was achieved with LISA processing parameters of 0.7 W and 0.7 m/s (Fig. 1b). The calculated maximal average thickens of copper deposition was in the order of *h*_max_ = *ρ*/*R*_s min_ ≈ 0.2 μm, where *ρ* = 1.68 × 10^−8^ Ω·m is the specific electrical resistivity of the copper^30^. The specific resistivity of electrolessly deposited copper thin films with the thicknesses up to 0.5 μm is 10–20 times higher than of bulk^31^. So, in our deposition case, the thickness of copper was in the sub-micrometer range. The absorption depth of copper in the visible range of the light spectrum is tens of nanometers^32^,^33^,^34^,^35^ and is the order of magnitude smaller than the thickness of deposited copper layer. Therefore, the colour was not influenced by the thickness of the metal layer.

### Percolation model of the sheet resistance

The conductivity of the polymer and metal mixture is divided to the three regions according to the percolation model^36^,^37^,^38^:


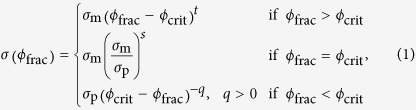


where *ϕ*_frac_ is the surface fraction of metal plated polymer surface; *ϕ*_crit_ is the percolation theshold when the conductivity is tending to zero; *σ*_m_ and *σ*_p_ are the conductivities of the metal and polymer constitutuents respectively; *t*, *s* and *q* are the exponental factors that depend on the dimesnioanlity of the system. The percolation model used to describe optical and electronic properties of nano-porous layers^39^,^40^,^41^ then can be generalized for all range of area fraction *ϕ*_frac_ values: below, above and at the percolation threshold. Therefore, the sheet resistance *R*_s_ is a function of *ϕ*_frac_:


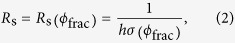


where *h* is the layer thickens of conducor and insulator mixture.

### Area fraction of copper dependence on the colour difference

**Figure 2:**
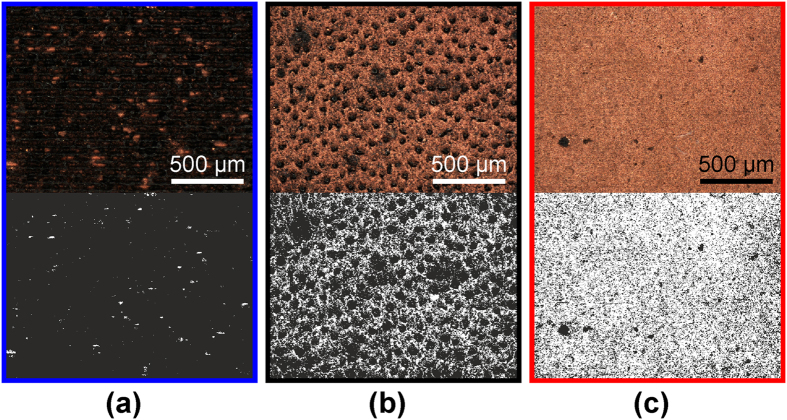
Porous copper on polymer. The dark field optical microscope images of deposited copper on the PC/ABS polymer (top row) and conversion of images to black and white mode at the threshold value of the luminance of *Y*_th_′ = 0.27 (bottom row). The naked polymer is given by the black colour and copper deposition by the white colour. The three different copper plating quality cases: (**a**) poor plating with *R*_s_ = 4300 Ω/sq, *ϕ*_frac_ ≈ 0.03, Δ*E* = 0.06; (**b**) moderate plating with *R*_s_ = 3.1 Ω/sq, *ϕ*_frac_ ≈ 0.65, Δ*E* = 0.44; (**c**) good plating with *R*_s_ = 0.14 Ω/sq, *ϕ*_frac_ ≈ 0.90, Δ*E* = 0.62.

**Figure 3:**
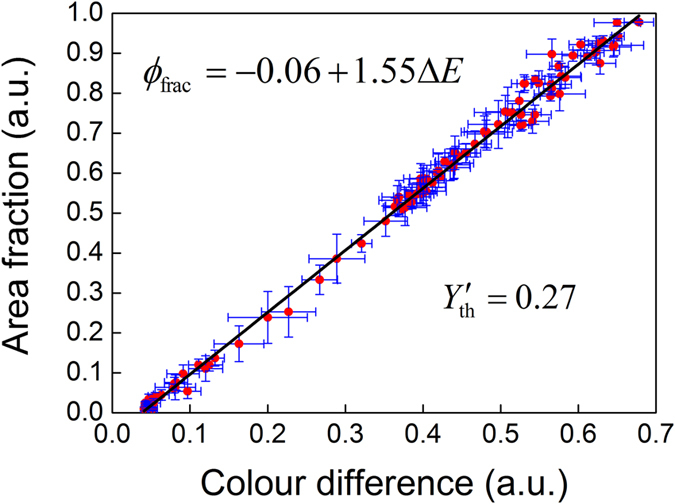
Area fraction vs. colour difference. Red circles (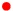) the measured area fraction of copper versus the colour difference between images of the non-plated and copper plated PC/ABS polymer surface. The area fraction *ϕ*_frac_ was calculated by using Equation (5) with the threshold value of the luminance *Y*_th_′ = 0.27. The colour difference Δ*E* was calculated by using Equation (4). The horizontal and vertical blue error bars indicate the standard deviation in the colour-difference and area fraction measurements taken from 5 sections of the microscope images, respectively. Black solid line is the linear least square fit of the experimental data points.

The obtained colour of the sample was affected by the fraction of the surface area plated by the metal *ϕ*_frac_. Openings in the polymer surfaces presented the colour of laser structured polymer. The copper covered part of the polymer produced the colour of copper (Fig. 2). In the case of a poorly plated surface, the copper plated areas did not have interconnection with each other, and, therefore, it possessed small average colour difference and high sheet resistance (Fig. 2a). The moderate plating quality represents the case when more than a half of polymer surface is covered by the metal islands and they have continuous net of interconnections between them through all the surface area (Fig. 2b). The well plated case appears when almost all surface area of the polymer is covered by the metal deposition and only small part of polymer is left open (Fig. 2c). The measured area fraction *ϕ*_frac_ of the copper-plated polymer versus the colour difference Δ*E* of the non-plated and plated surface is given in Fig. 3. In the particular case with the threshold value of 0.27 the area fraction had linear dependence on the colour difference (Fig. 3):


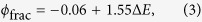


Similar linear dependence has been reported for the semi-transparent copper nanowire film in the area fraction versus transmittance representation by J. W. Borchert *et al.*^18^ and by S. M. Bergin *et al.*^19^. However, in the general case our measured fraction of the sample surface covered by the metal was a function of the colour difference and the threshold value of luminance as 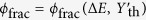. The measurement of the area fraction is sensitive to the threshold value *Y*_th_′, and obtains linear relationship only with particular the value of 0.27. The determination of the correct threshold value requires additional calculations. The way to avoid this is to use colour difference instead of area fraction. Taking in to accout that 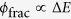, the percolation model of electrical conductance of isolator-conductor mixture can be adapted to our experimental conditions. The sheet resistance from Equations (1, 2, 3) becomes a function of colour difference *R*_s_ = *R*_s_(Δ*E*).

### Sheet resistance dependence on the colour difference

**Figure 4:**
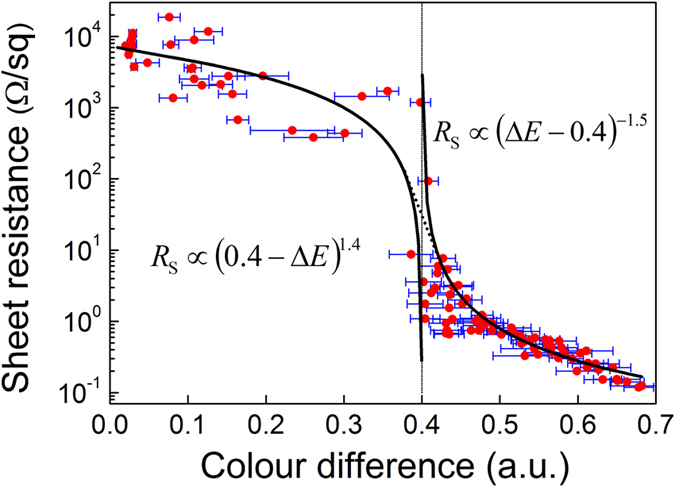
Sheet resistance vs. colour difference. Red circles (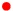) represent the measured sheet resistance of the copper-plated areas versus the colour difference between images of the non-plated and copper plated PC/ABS polymer surface. The colour difference Δ*E* was calculated by using Equation (4). Black solid lines are the fits of experimental data points by the percolation model Equations (1, 2, 3). The blue horizontal error bars indicate the standard deviation in the colour-difference taken from 5 sections of the microscope images.

The sheet resistance *R*_s_ of the metal-plated polymer areas versus the colour difference Δ*E* of the non-plated and plated surface is given in Fig. 4. The sheet resistance of copper on the polymer surface has a power law dependence on the colour-difference of the non-plated and plated areas described by the percolation model Equations (1, 2, 3). The exponents *q* = 1.4, *s* = 0.5 and *t* = 1.5 of the power law in the generalized percolation model of sheet resistance were found from the fits of the experimental data for poorly, moderately and well plated areas, respectively (Fig. 4). The decrease of sheet resistance with the colour difference occurs because the higher is the amount of copper deposited on the sample, the bigger the colour difference is, and therefore, results the lower sheet resistance. This power law relationship can be converted to the quantity of copper plated onto the polymer. In this way, the quality of copper plated areas by the ECP procedure can be evaluated from optical microscope images, by simple calculation of colour difference.

The colour difference calculations have been performed by using the five major models of the colour spaces: International Commission on Illumination Lightness a-chroma b-chroma (CIELab); Red Green Blue (RGB); Luma Plus Chroma (YUV); Hue Saturation Value (HSV); Cyan Magenta Yellow Key (CMYK). The identical results in sheet resistance versus colour difference representation were achieved independently on the colour space used. The RGB colour metric has been chosen for the simplicity because it does not require any conversion between colour spaces of the images taken by CCD camera.

After a period of two months the same colour measurement procedure was carried out with the same particular sample. No significant changes of the colour of copper plated samples were observed.

### Optimal processing conditions for best plating quality

**Figure 5:**
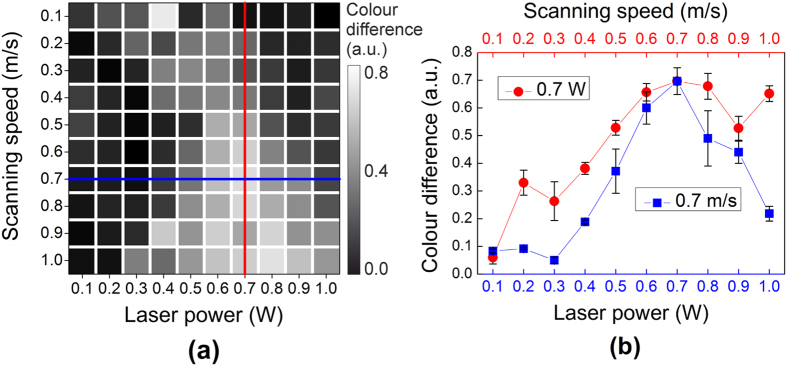
Colour difference vs. processing parameters. (**a**) The map of colour difference between images after the LISA and ECP procedures at different laser powers and scanning speeds. (**b**) The colour difference versus scanning speed at 0.7 W laser power - red circles (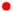); colour difference versus laser power at 0.7 m/s scanning speed - blue squares (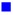). Colour difference was calculated by using Equation (4). The vertical error bars in (**b**) indicate the standard deviation in the colour-difference measurements taken from 5 sections of the microscope images.

The map of the colour difference of all processed areas was plotted in Fig. 5a. The colour-difference map (Fig. 5a) is in good agreement with the colour of samples after the ECP procedure (Fig. 1b). The minimal sheet resistance of 0.1 Ω/sq was achieved by using laser the power of 0.7 W and the scanning speed of 0.7 m/s. This point with the best plating quality corresponds well to the maximum measured colour difference of 0.7 (Fig. 5b).

## Conclusions

To summarise, the PC/ABS polymer was structured by using different laser processing parameters by the LISA method. The laser-structured areas were selectively plated by the copper layer by using the ECP procedure. The colour difference of the sample images was measured before and after metal deposition. The sheet resistance of copper layers decay by the power-law with increasing of the colour difference. The percolation theory of electrical conductance of insulator-conductor mixture has been adopted in order to explain the experimental results. Three distinguishable regions of sheet resistance decay correspond well with the generalized percolation model. The quality of copper plating on polymers depended on both processing parameters: the laser power and the scanning speed. A new colorimetric method is an easy tool for evaluation of the metal plating quality requiring only an optical microscope or a colorimeter. The new proposed method has been proven to be independent on the colour space used for the colour difference measurements.

## Methods

### Material

A polycarbonate/acrylonitrile butadiene styrene (PC/ABS) polymer, commonly used in moulding of electronic components, was selected as the test material for the LISA and ECP procedures in this work.

### Laser Induced Selective Activation (LISA) Procedure

**Figure 6:**
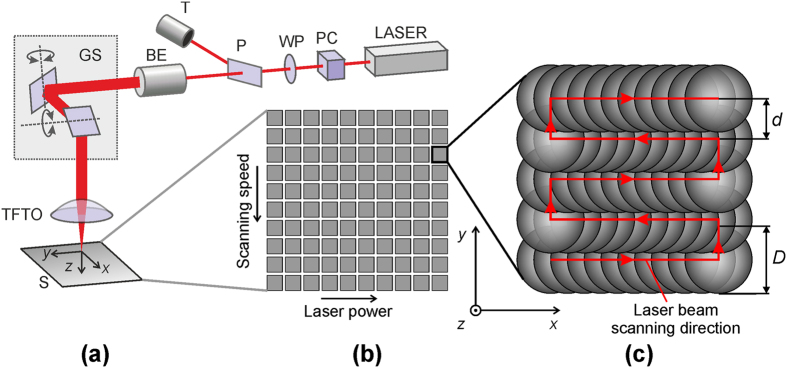
Experimental setup and procedure for LISA. (**a**) Experimental setup: LASER: a nanosecond laser, PC: Pockels cell, WP: half wavelength wave plate, P: polarizer, T: trap, BE: beam expander, GS: galvanometer scanner, TFTO: telecentric f-theta lens. (**b**) Schematic representation of an array of laser scanned squares on the polymer sample with variable processing parameters: the laser power and the scanning speed. (**c**) Schematic representation of the laser beam scanning pattern in each of the squares: red step-type line represents the path of the scanned laser beam on the sample, red arrows - the beam scanning direction, grey circles - overlapped laser spots, *d* - is the hatching distance, *D* - is the laser spot diameter.

The LISA procedure consisted of a single step: the direct laser structuring of the polymer surface. It was performed in order to induce the porous sponge-like structures to the surface of the polymer. Experimental setup for LISA of the polymer is shown in Fig. 6a. The Q-switched diode pumped solid state nanosecond Nd:YVO_4_ laser (Baltic HP, Ekspla) was used in the experiments. The main parameters of the laser were: the pulse duration 10 ns; the pulse repetition rate 50 kHz; the wavelength of irradiation 532 nm; the mean laser power up to 1.0 W. The laser power was varied by using an external attenuator that consisted of the Pockells cell, a half wavelength wave plate, the polarizer and a trap. The beam expander was used to change the beam diameter and adjust a spot size on the sample. The laser beam spot on the sample was scanned by using a galvanometer scanner (hurrySCAN14, SCANLAB) equipped with a telecentric f-theta lens with the focal length of 80 mm at the scanning speed of up to 1.0 m/s.

The 10 × 10 arrays of square segments, each size of 3 × 3 mm^2^ were written on the polymer samples by scanning the parallel partly-overlapping lines by changing processing parameters (mean laser power and scanning speed) for each square Fig. 6b. The laser power was varied from left to right from 0.1 W by 0.1 W to 1.0 W and the scanning speed was changed from top to bottom from 0.1 m/s by 0.1 m/s to 1.0 m/s. Each square was patterned by hatching the laser beam spot as shown in Fig. 6c. The hatching distance between each scanned line was *d* = 50 μm. The laser spot size was adjusted to *D* ≈ 100 μm by changing a magnification factor of the beam expander. Scanning of each square was repeated 10 times in the order to induce sufficient porosity of the polymer surface as recommended in work by Y. Zhang *et al.*^9^. The laser surface treatment of the polymer surface was performed at room temperature in air. Later, the copper layer was deposited by the ECP procedure selectively only onto the laser structured areas keeping unstructured areas free of the metal deposition.

### Electroless Copper Plating (ECP) Procedure

The ECP procedure consisted of three-steps. Firstly, the laser-patterned samples were immersed in the palladium colloidal activator solution SnCl_2_/PlCl_2_ for 5 min. Secondly, cleaned with distilled water. Thirdly, they were immersed in the electroless copper plating bath: CuSO_4_ × 5 H_2_O (0.12 M), KNaC_4_ H_4_O_6_ × 4H_2_O (0.35 M), NaOH (1.25 M), Na_2_CO_3_ (0.3 M), HCOH (3.41 M) for 10 min. The samples were dried at normal conditions: in the air at the room temperature.

### Colour Difference Measurement

The colour difference or distance measurements were performed by using an optical microscope (Eclipse LV100, Nikon) equipped with the high-definition 5-megapixel CCD camera (DS-Fi1, Nikon) with the resolution of 2560 × 920 pixels. The digital camera was controlled by the microscope camera controller (Digital Sight DS-U2, Nikon) and the imaging software (NIS-Elements D, Nikon). The microscope objective (LU Plan Fluor 5×, Nikon) with the magnification factor of 5× and the numerical aperture of 0.15 was used in the dark field mode. The illumination source of the microscope consisted of a 50 W halogen lamp (LV-HL50PC, Nikon). All the microscope apertures were fully opened in order to provide maximal available sample illumination. The white balance was performed by using a sheet of white paper and the red, green, and blue component coefficients *R*_w_ = 1.64, *G*_w_ = 1.00 and *B*_w_ = 1.98 were set for the colour measurement experiments. The exposure time of 10 ms and the gain factor of 1.7 were determined by the auto exposure mode and later these factors were used in the manual exposure mode. The RGB images with the 8-bit colour depth, the size of 640 × 480 pixels and the BMP file format were taken averaging the 4 × 4 pixel area of CCD camera into the one pixel. The actual size of each photographed area was 1.74 × 1.31 mm^2^. All samples were photographed before and after copper deposition. The pictures were divided into 5 sections with the size of 128 × 480 pixels. The average colour was calculated for each section. The colour difference Δ*E* was calculated between images after the ECP (after copper deposition) and LISA (before copper deposition) for each of the five sections by using an equation:


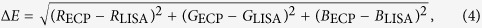


where *R*_ECP_, *G*_ECP_, *B*_ECP_, *R*_LISA_, *G*_LISA_, *B*_LISA_ are average red, green and blue components of the sample images after the ECP and LISA procedures. The average colour difference and the standard deviation of it were calculated from the data achieved from five sections. The digital processing of the images and colour difference calculations was performed by computer algebra system software (Maple, Maplesoft).

### Copper area fraction measurement

Digital RGB optical microscope images of copper plated areas were converted to the grayscale mode by using formula based on the NTSC standard^42^:


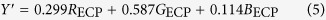


where *Y*′ is the luminance of the image after ECP procedure. The area fraction *ϕ*_frac_ of the copper was evaluated by calculating the ratio of number of pixels above certain threshold *Y*_th_′ to the total number of the picture pixels. The average area fraction and the standard deviation of it were evaluated from the five sections of the image.

### Sheet Resistance Measurement

The Kelvins four-terminal sensing technique also called four-point collinear probe method was used to measure the sheet resistance of the copper-plated areas on the polymer sample^43^,^44^. The linear array of four-point-probes with the needles separated by the probe spacing distance of 0.5 mm was used in the experiments. The sheet resistance was measured by using a source meter (2602 A, Keithley) and the measurement software (TSP^®^ Express, Keithley).

## Additional Information

**How to cite this article**: Gedvilas, M. *et al.* Colour-Difference Measurement Method for Evaluation of Quality of Electrolessly Deposited Copper on Polymer after Laser-Induced Selective Activation. *Sci. Rep.* **6**, 22963; doi: 10.1038/srep22963 (2016).
